# Giant genome of the vampire squid reveals the derived state of modern octopod karyotypes

**DOI:** 10.1016/j.isci.2025.113832

**Published:** 2025-10-23

**Authors:** Masa-aki Yoshida, Emese Tóth, Koto Kon-Nanjo, Tetsuo Kon, Kazuki Hirota, Atsushi Toyoda, Hidehiro Toh, Hideyuki Miyazawa, Makoto Terauchi, Hideki Noguchi, Davin H.E. Setiamarga, Oleg Simakov

**Affiliations:** 1 Marine Biological Science Section, Education and Research Center for Biological Resources, Faculty of Life and Environmental Science, Shimane University, Oki, Shimane 685-0024, Japan; 2 Department for Neurosciences and Developmental Biology, University of Vienna, Universitätsring, Vienna 1030, Austria; 3 Department of Applied Chemistry and Biochemistry, National Institute of Technology (KOSEN), Gobo, Wakayama 644-0023, Japan; 4 Graduate School of Science, The University of Tokyo, Bunkyo-ku, Tokyo 113-0033, Japan; 5 Advanced Genomics Center, National Institute of Genetics, Mishima, Shizuoka 411-8540, Japan; 6 Center for Genome Informatics, Joint Support-Center for Data Science Research, Research Organization of Information and Systems, Mishima, Shizuoka 411-8540, Japan; 7 Advance School of Ecosystem Engineering, National Institute of Technology (KOSEN), Wakayama College, Gobo, Wakayama 644-0023, Japan; 8 Institute for Community Innovation in Collaboration with KOSEN (MILLA), Toyohashi University of Technology, Toyohashi, Aichi 441-8580, Japan

**Keywords:** Marine organism, Genetics, Evolutionary mechanisms, Phylogenetics, Genomic analysis

## Abstract

Ancient evolutionary transitions in animal chromosomal complements and their phenotypic impacts remain understudied. Few systems exist where these events can be dissected into individual steps. In coleoid cephalopods (octopus, squid, cuttlefish), an ancient coleoid chromosomal rearrangement event (ACCRE) resulted in a substantial increase in the chromosome number. However, the discrepancies between extant octopodiform (octopus, ∼30 chromosomes) and decapodiform (squid and cuttlefish, ∼46 chromosomes) karyotypes and the direction of this transition remain unexplained. Through sequencing of the basally branching octopodiform, the vampire squid *Vampyroteuthis* sp., we reveal its partial retention of the decapodiform karyotype. Together with the chromosome-level assembly of the pelagic octopod *Argonauta hians*, we show that modern octopod genomes were extensively shaped by chromosomal fusion-with-mixing followed by inter-chromosomal translocations. These irreversible processes have resulted in a more entangled genomic configuration in octopods. Our results offer broader insights into general patterns of chromosomal evolution following large-scale rearrangement in animal genomes.

## Highlights

• The *Vampyroteuthis* genome is one of the largest animal genomes, exceeding 10 Gbps
• Vampyroteuthis genome retains karyotype features of squids and cuttlefishes
• Karyotype reduction and rearrangements occurred in octopod genomes
• Syntenic pattern in octopods suggests another period of intense rearrangements

## Graphical abstract

**Figure:**
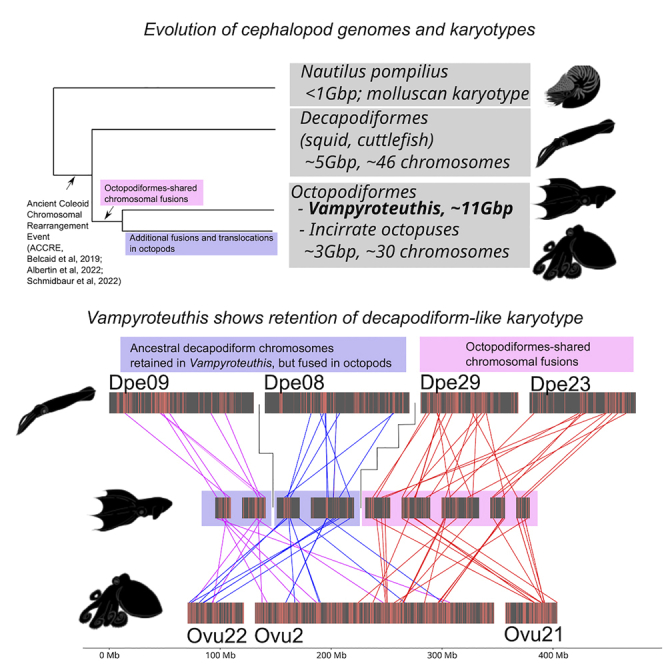

## Introduction

Cephalopods (octopuses and squids) are known for their complex behaviors,^1^ including problem solving, learning by observation, executing different tasks, and exceptional camouflaging ability.^1^ Although similar in size to those of mammals, coleoid nervous systems are structurally distinct and have a long evolutionary history associated with advanced cognition and neural complexity.

**Figure 1:**
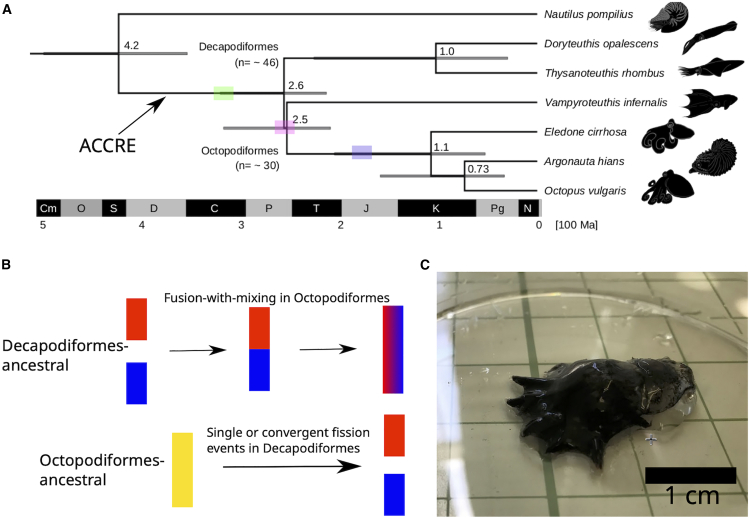
Early origins of coleoid cephalopods and conflicting chromosome evolution scenarios (A) Phylogenetic time tree of mitochondrial genomes with the major coleoid cephalopod clades and their closest outgroups. Node branches are labeled according to the major cephalopod evolutionary transitions. Node bars show the confidence intervals of the divergence times. (B) Two conflicting scenarios how coleoid karyotypes evolved, reduction and expansion (via fission) scenarios. Fusion-with-mixing (FWM) provides a powerful synapomorphic character to profile irreversible changes. (C) *Vampyroteuthis* specimen used for sequencing. Scale bar represents 1 cm. ACCRE: ancient coleoid chromosomal rearrangement event.

Cephalopods split into nautiloids and the lineage leading to coleoids around the Late Silurian (ca. 422 Ma)^2^ (Figure 1A). While extant nautiloids retained their external shells, coleoids internalized or lost theirs, enabling greater maneuverability and adaptation to diverse marine environments.^3^^,^^4^ Shell loss may also have driven the evolution of sophisticated neural processing leading to adaptive and complex behaviors such as rapid decision-making and camouflaging.^5^ By the Late Permian to the Middle Triassic (∼260–235 Ma), coleoids had further diversified into Octopodiformes (vampire squids and octopuses) and Decapodiformes (squids and cuttlefishes)^2^^,^^6^^,^^7^^,^^8^ (Figure 1A).

The evolution of behavioral and neural complexities in coleoids, which was probably driven by clade-specific predation pressures and ecological adaptations,^9^^,^^10^^,^^11^^,^^12^ likely required major genetic innovations. Recent studies have begun to uncover how large-scale changes in genome structure contributed to the evolution of their neural, cognitive, and other traits unique to coleoids.^13^^,^^14^^,^^15^^,^^16^ Comparative analyses show coleoids differ substantially in genome organization from other mollusks.^13^^,^^14^ While extant nautiluses retain the typical molluscan genomic structure,^16^^,^^17^^,^^18^ coleoids underwent “ancient coleoid chromosomal rearrangement event^15^^,^^19^ (ACCRE)”, causing topological reorganizations that restructured gene linkages and formed novel regulatory landscapes.^18^

While ACCRE marked a key genomic transition in early coleoids, substantial lineage-specific changes between major coleoid lineages likely happened post-ACCRE. Octopuses and squids differ in chromosome numbers,^15^ repeat content,^19^ and expansions of gene families such as *Protocadherins*^20^ and *GPCRs*.^21^ Interestingly, while coleoid chromosomes have remained largely intact within both decapodiform and octopodiform lineages, their homologies are not strictly one-to-one, with some chromosomes showing many-to-many correspondences.^15^ The discrepancy between predicted typical chromosome numbers (∼30 in Octopodiformes vs. ∼46 in Decapodiformes) also raises questions about whether chromosome numbers contracted or expanded during early coleoid evolution (Figure 1B). The lack of genomic data from early branching Octopodiformes or Decapodiformes has obscured the directionality of these transitions.

The vampire squid (*Vampyroteuthis* sp.), a basally branching Octopodiformes, provides critical insight into the deep evolutionary history of coleoids.^22^ This species is a globally distributed deep-sea pelagic cephalopod with an opportunistic detritivorous and zooplanktivorous feeding strategy.^23^^,^^24^ This “living fossil” is the only extant representative of the vampyropods, an ancient clade within Octopodiformes (Figure 1) that has existed since the Mesozoic Era, from at least the Middle Jurassic (e.g., *Vampyronassa*; ca. 165 Ma)^24^^,^^25^ with some evidence suggesting its origins in the Early Jurassic (e.g., *Teudopsis* and *Simoniteuthis*; ca. 183 Ma).^25^^,^^26^^,^^27^ Ancient vampyropods coexisted with the ammonites and the belemnites throughout the Jurassic (∼201–145 Ma) and Cretaceous (∼145–66 Ma), occupying various ecological niches across global marine environments.^26^^,^^28^^,^^29^ However, most vampyropod lineages went extinct at the end of the Cretaceous (∼66 Ma), eventually leaving extant *Vampyroteuthis* as the sole surviving species.^30^ *Vampyroteuthis*’ unique phylogenetic position makes it a valuable model for resolving early genomic transition events and subsequent evolutionary trajectories in Octopodiformes and coleoids.

To enhance resolution within Octopodiformes, we also present the chromosome-level genome assembly of the muddy argonaut *Argonauta hians*, a member of Argonautoidea, an octopodiform clade composed mainly of pelagic octopods characterized by the presence of the secondarily acquired shell-like calcified eggcases.^31^^,^^32^ The inclusion of these two genomes in our analyses enables us to reveal the directionality and trajectory of chromosomal rearrangements, genomic reorganizations, and size variation across early and modern cephalopods.

## Results

We sequenced the draft genome of *Vampyroteuthis* sp. from an individual collected in the West Pacific Ocean. Genome sequencing was performed using the PacBio HiFi platform at approximately 40X coverage and assembled with hifiasm, resulting in a 14 Gb assembly. Different assembly settings were tested, yielding similar assembly sizes (Table S1). A more stringent setting to both haplotype overestimation and to remove duplicates still resulted in similar completeness (BUSCO score of 95%) and an assembly size of over 11 Gb (Table S3). Therefore, regardless of the assembly settings, the total length estimates confirm that the *Vampyroteuthis* genome constitutes the largest cephalopod genome sequenced to date (Figure S1; and Table S2). Since we could not currently obtain additional samples for this rare deep-sea species, chromosomal conformational data are not yet attainable. However, while our *Vampyroteuthis* draft genome assembly consists of numerous sub-chromosomal level contigs (about 5,600 contigs, N50 of 9.96 Mb, and the longest contig of 51.7 Mb), it still contains sufficient syntenic information,^33^ with 162 contigs containing 15 or more orthologous genes and the largest contig containing 130 orthologous genes. Meanwhile, the size of the genome of *Argonauta hians* was ∼1.52 Gb, similar to that of its congener *A. argo*,^32^ but only half of that of *Octopus* (∼3 Gb).^14^ The draft genome of *A. hians* was assembled into 28 putative chromosomes, comparable in contiguity and resolution to other octopod genomes, which typically have 30 chromosomes (Figure S2).

**Figure 2:**
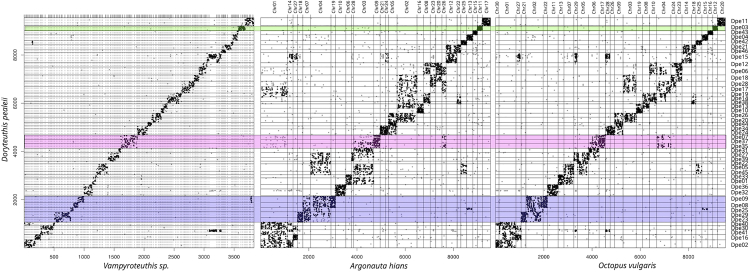
*Vampyroteuthis* genome suggests ancestral coleoid karyotype was decapodiform-like, followed by karyotype reduction in Octopodiformes Synteny dotplots showing Octopodiformes and Argonauta-Octopus shared FWM characters, relative to the *Doryteuthis pealeii* chromosomes. *Doryteuthis* chromosomes are ordered in the same way across the three species comparisons. The color codings indicate representative examples of the ancestral coleoid chromosome (green), ancestral Octopodiformes mixed chromosome (pink), and fused-and-mixed chromosomes in octopods (blue), respectively (corresponding to the phylogenetic nodes highlighted in Figure 1).

Gene mixing on fused chromosomes is irreversible, and thus any ancestral or fusion-with-mixing (FWM) would serve as a strong synapomorphy for the clade in question.^34^^,^^35^ To investigate how genome architectures relate to chromosomal evolution, we conducted comparative analyses across multiple cephalopod lineages using a modified synteny approach (see STAR Methods). This approach enabled us to profile the presence of chromosomal irreversible FWM (Figure 2) and to confirm the highly rearranged nature of coleoid chromosomes relative to the nautilus (Figure S3).^16^ Despite its gigantic size due to massive expansion of repetitive elements (62% or at least 6 Gb of the genome, Figure S4), we also found that the *Vampyroteuthis* genome structure predominantly adheres to basic coleoid karyotype rather than the more ancestral and small sized nautilus genome (730.5 Mb) or the general molluscan chromosomal complements (Figure S3).^15^ This pattern remained consistent even when alternative mapping and orthology search strategies were applied (Figure S4), confirming the robustness of this result.

**Figure 3:**
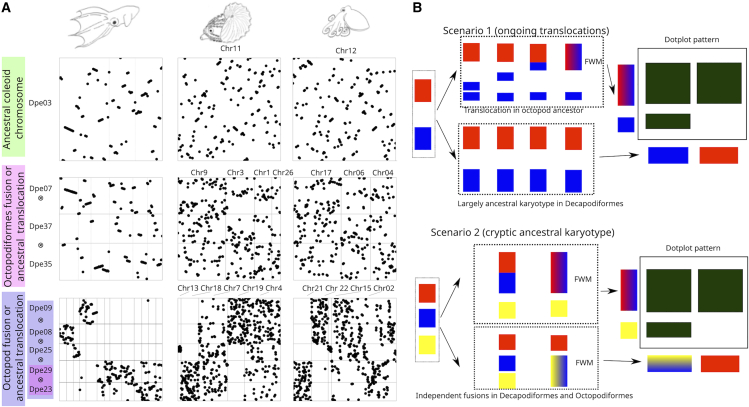
Chromosomal evolutionary pattern suggests additional ancestral translocations in octopods (A) Dotplot representation for the three cases highlighted in Figure 1, with the *y* axis indicating the chromosomes of *Doryteuthis*. Green indicates conserved coleoid chromosomes, as suggested by the conservation of genes in the chromosomes of all compared coleoids, despite translocation of the genes’ location within the chromosomes (intrachromosomal rearrangements; “mixing in a chromosome”). Meanwhile, ancestral Octopodiformes (pink) indicate fusions of different *Doryteuthis* chromosomes, which happened in ancestral Octopodiformes. The third example (blue) shows the formation of complex patterns of fusion-with-mixing, where orthologous genes undergoing intrachromosomal translocations in specific regions of the chromosomes of *Doryteuthis* are present in multiple chromosomes in compared species. (B) Two main scenarios for the observed complex chromosomal evolutionary patterns. The first one involves a fusion-with-mixing of intact (in red) and partial (in blue) chromosomes following inter-chromosomal translocations (blue) that occurred in Octopodiformes after divergence from the decapodiform karyotypes. A second possible scenario suggests that following the split of Octopodiformes and Decapodiformes, each lineage experienced distinct fusion-with-mixing events involving multiple ancestral chromosomes. In Octopodiformes, two specific chromosomes fused and mixed (red and blue), while in Decapodiformes, a different pair of chromosomes (blue and yellow) underwent a similar FWM process.

Surprisingly, we found that the *Vampyroteuthis* chromosomal structure is more closely aligned with that of Decapodiformes, including those of the longfin squid *Doryteuthis pealeii*, the bobtail squid *Euprymna scolopes*, the diamond squid *Thysanoteuthis rhombus*, and the cuttlefish *Sepia lycidas* (Figures 2 and S5), despite its well-supported phylogenetic placement in Octopodiformes and thus its affinities with the octopods. Although our *Vampyroteuthis* draft genome assembly is not at the chromosomal level, its contigs often correspond to single *Doryteuthis* chromosomes, reflecting a high degree of chromosomal retention. Conversely, *Vampyroteuthis* contigs exhibited complex relationships with the chromosomes of the other octopodiform species (*A. hians*, the curled octopus *Eledone cirrhosa*, and the common octopus *Octopus vulgaris*), indicating synapomorphic fusions and rearrangements shared across octopods (Figures 2 and S5). Multiple *Doryteuthis* chromosomes and their one-to-one homologous *Vampyroteuthis* sets of contigs correspond to single octopod chromosomes and show highly entropic mixing. For example, *O. vulgaris* chromosome 22 corresponds to *Doryteuthis* chromosomes 8 and 9, each with its own non-overlapping set of *Vampyroteuthis* contigs (Figure 3A), with the degree of entropic mixing of these two states along the *Octopus* chromosome of 0.193, as measured by the normalized turbulence.^36^ The quartile range of normalized turbulence for all fused chromosomes in our dataset between *O. vulgaris* and *D. pealeii* (Data S1) was between 0.105 and 0.209, with higher value indicating more mixing. This implies that FWM of two ancestral decapodiform-like chromosomes (*Doryteuthis* chromosomes 8 and 9) occurred at the base of the octopod (*Eledone*, *Argonauta*, *Octopus*) lineage after its separation from *Vampyroteuthis*. For comparison, *O. vulgaris* chromosome 2 (Figure 3A) shows inter-chromosomal translocations with sharp syntenic boundaries and little mixing (0.081 normalized turbulence for *Doryteuthis* chromosomes 9 and 29, below the quartile range), suggesting a more recent origin of this translocation event. These chromosomal changes most likely accumulated gradually in the lineage leading to the octopods after their divergence from the *Vampyroteuthis* lineage (Figures 2 and S5). Given the basal position of *Vampyroteuthis* among the Octopodiformes, the conservation of chromosomal structure across decapodiform lineages and *Vampyroteuthis*, along with the drastic chromosomal rearrangements shared by all octopods, suggests the ancestral nature of the decapodiform karyotype.

To further explore the extent of chromosomal fusion and mixing in the lineage leading to octopods, we examined how *Doryteuthis* chromosomes were mapped onto the *Vampyroteuthis* genome. The results revealed chromosomal rearrangements with a high degree of mixing within the *Vampyroteuthis* contigs. For example, contig ptg000753l is mapped to *Doryteuthis* chromosomes 7, 37, and 35 with normalized turbulence measure of 0.285 for Dpe35 and Dpe37, 0.290 for Dpe07 and Dpe37, and 0.193 for Dpe07 and Dpe35.^36^ The pattern is observed for 33 out of the 46 *Doryteuthis* chromosomes, where parts of the chromosomes were mapped onto the same set of *Vampyroteuthis* contigs (Fisher’s exact test *p* value <0.05), suggesting irreversible FWM. As the same chromosomes were found to be fused and mixed in all other octopodiform species, this suggests an ancient synapomorphy supporting the affinity of *Vampyroteuthis* with octopods. On top of this synapomorphic signature, further substantial chromosomal FWM occurred in the lineage leading to octopods (*Eledone*, *Argonauta*, and *Octopus*) (Figure 2). Octopod homologs of only 3 *Doryteuthis* chromosomes (including the proposed sex chromosome^37^) remain unfused, suggesting that 43 out of 46 chromosomes underwent fusions on the branch leading to octopods. Of these, at least 10 chromosomes remain unfused in *Vampyroteuthis*, indicating additional fusions after the divergence of octopods from the vampyropod lineage. Comparison with the *O. vulgaris* karyotype showed that the argonaut underwent further fusions between chromosomes 3 and 8, as well as 6 and 7, reducing the chromosome number from 30 to 28, suggesting a general trend of chromosomal number reduction in octopuses.

Our observations strongly suggest that the ancestral coleoid had a decapodiform-like karyotype, which later underwent additional fusions and karyotype reduction, first at the base of Octopodiformes, and then later in the octopods (Figure 2). The alternative scenario of chromosomal fissions is highly unlikely, as it would require independent, convergent fissions of hundreds of genes to produce identical chromosomal splits observed in all Decapodiformes and *Vampyroteuthis*.^35^ We could also confirm this pattern by partitioning the *O. vulgaris* genome into fragments that contain the same gene content as those found in our *Vampyroteuthis* assembly. Using the same syntenic analysis approach, this artificially and fragmented *Octopus* genome was still recapitulating octopod fusions (Figure S6), besides also showing a signal for the fusion of 42 out of 46 *Doryteuthis* chromosome homologs, compared to the 43 fused *Doryteuthis* chromosome homologs found in the complete *O. vulgaris* genome (Fisher’s exact test *p* value <0.05). This suggests that the contiguity of the *Vampyroteuthis* assembly is unlikely to impact the inference of chromosomal fusions. These results suggest that *Vampyroteuthis* has a chromosomal structure partially shared with extant Decapodiformes, supporting the interpretation that it retains the more ancestral coleoid configuration, while octopods underwent additional FWMs.

We also observed a complex syntenic pattern in octopod chromosomes that cannot be explained by simple FWMs (Figure 3). Two possible scenarios might explain this complex syntenic pattern (Figure 3B). First, it may suggest that major inter-chromosomal translocations have occurred in the lineage leading to octopods (*Eledone*, *Argonauta*, and *Octopus*; Figure 3B). The second possibility is that a large post-ACCRE ancestral coleoid karyotype independently fused into different configurations in Decapodiformes and Octopodiformes (Figure 3B). If the second scenario was the case, we would expect to find a decapodiform species with an unfused chromosomal pair that corresponds to separate chromosomes in *Vampyroteuthis* or octopods yet fused in other Decapodiformes. However, since we have yet to find such decapodiform species, our data most strongly support the first scenario (Figure S7). Although similar syntenic patterns can also arise after whole genome duplication,^35^ we found no evidence of paralogous enrichment on any of the octopodiform chromosomes. Besides that, the observed chromosomal correspondence pattern does not indicate the presence of a consistent genome-wide doubling of ancestral chromosomes. Therefore, we believe it is unlikely for any whole-genome duplication event to have happened and be the cause of the syntenic patterns we observed. Additionally, comparisons within the octopods (Figure S8) show that *Eledone*, despite its basal position on the tree, showed additional modifications to its karyotype, including translocations to the otherwise highly conserved ancestral coleoid chromosomes Dpe03-Ovu12. On the other hand, genomes of *A. hians* and *O. vulgaris* were strikingly similar and shared more chromosomal characteristics with *Vampyroteuthis*, suggesting a more ancestral octopod karyotype (Figure S8).

Why has the vampire squid maintained its more ancestral chromosomal state despite a dramatic increase in genome size? While several studies^38^^,^^39^^,^^40^ have implied the effects of transposable elements (TEs) accumulation on enhancing genome rearrangement rate, some of the largest genomes sequenced to date (e.g., lungfish^41^) show surprisingly well-conserved karyotypes. The *Vampyroteuthis* genome provides another example where TE-driven genome expansion was decoupled from an increase in genome rearrangements. Future studies of both epigenetic states and genome topology in this species should provide fruitful insight into the role of TE accumulation in the maintenance of ancestral genomic configuration in animal genomes.

**Figure 4:**
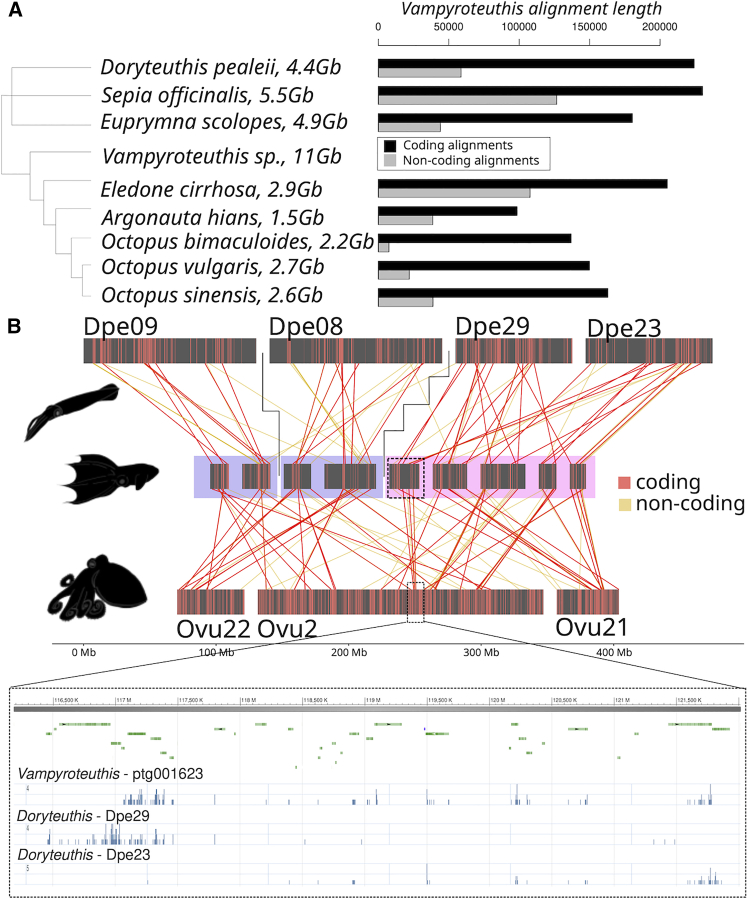
Conserved non-coding element complement in coleoid genomes (A) *Vampyroteuthis*-centered whole genome alignments show the total content (in alignment numbers) of coding and non-coding alignments. (B) Ribbon diagram showing location of homologous alignments of coding (red, CDS) and non-coding (orange) regions between *Doryteuthis* chromosomes (top) to *Vampyroteuthis* (middle) and *Octopus vulgaris* (bottom) for the complex FWM shown in Figures 2 and 3 (pink and blue colors). Zoom-in on a middle portion of *O. vulgaris* chromosome 2 (Ovu2) is highlighted below (NCBI genome browser) with gene track of annotation and conservation of both coding and non-coding elements derived from one homologous *Vampyroteuthis* contig and its pre-octopodiform unmixed state on two chromosomes Dpe29 and Dpe23 (pink color). Two *Vampyroteuthis* contigs homologous to Dpe09 and Dpe08, on the other hand, remain unmixed and their homologs undergo FWM only in octopods (blue color).

To further investigate the role of chromosomal fusion events onto putative regulatory landscapes as reflected by conserved non-coding element (CNE) preservation, we have conducted whole genome alignments between *Vampyroteuthis* and other coleoid cephalopods (see STAR Methods). We found that the conservation of non-coding regions was higher between *Vampyroteuthis* and Decapodiformes (over 7.1 Mb aligned sequences) compared to *Vampyroteuthis* and *Octopus* (2.5 Mb total alignment, Figure 4A). The decrease in the overall alignment length is particularly visible in *Argonauta* and *Octopus*. This pattern, while correlated with the genome size, is also corroborated by the overall repeat composition of the *Vampyroteuthis* genome, with long interspersed nuclear elements (LINEs) comprising a major part of the genome (14.12%) and short interspersed nuclear elements (SINEs) less than 1% (Table S4). This is similar to other Decapodiformes genomes and in contrast to the SINE-dominated octopod genomes,^19^ indicating *Vampyroteuthis*’s higher propensity to retain ancestral non-coding features.

Using chromosomal homologies inferred from gene-level comparisons (Figures 2 and 3), we traced their CNE complement evolution on the derived octopod chromosomes (Figure 4B). *Vampyroteuthis* homologs of unfused *Doryteuthis* chromosomes (Dpe09 and Dpe08) remain unmixed as separate units, whereas *Vampyroteuthis* contigs that correspond to the predicted octopodiform FWM events show substantial mixing of both coding and non-coding elements (Dpe29 and Dpe23, Figure 4B). The observed peak patterns on Dpe29 and Dpe23 align within a single fused region in both *Vampyroteuthis* and *Octopus*, consistent with an irreversible fusion event. All these contigs map to a set of *O. vulgaris* chromosomes, resulting in a high degree of mixing of gene loci and their putative regulatory sites. From this observation, we can infer that a large portion of the regulatory landscapes on modern *O. vulgaris* chromosomes has a complex evolutionary origin, shaped by FWM events combining segments derived from distinct chromosomal units in the ancestral octopodiform lineage.

## Discussion

Our findings show that the decapodiform-like karyotype represents the ancestral state of coleoids and support the basal position of *Vampyroteuthis* within Octopodiformes. This conclusion aligns well with paleontological data and insights regarding cephalopod bauplan evolution. Stem coleoid species from the Paleozoic, such as *Gordoniconus* and *Bactrites*, exhibit internal shells, streamlined morphologies, and ten arms, long before the appearance of the oldest vampyropods in the Jurassic, such as *Teudopsis* and *Vampyronassa*. Interestingly, *Vampyroteuthis* also possesses two long feeding filaments, thought to be vestigial arms, indicating a possible morphological affinity to the 10-armed decapodiform. The retention of parts of the decapodiform-like chromosomal architecture in *Vampyroteuthis*, along with the extensive chromosomal rearrangements observed in octopods, provides genomic evidence supporting the transition from decapodiform-like ancestors to Octopodiformes. Our analyses also indicate a surge of inter-chromosomal translocations at the base of the octopods, potentially facilitated by demographic shifts or genomic instability. These ACCRE-like inter-chromosomal translocations, possibly stabilized by the notably large genome sizes in coleoids, may have driven morphological innovations and regulatory complexity. For instance, the specialization of arms into feeding tentacles in squids and the reduction of arms in octopuses likely result from modifications in gene regulation and its chromosomal context, and not gene content. Similarly, the presence of shell matrix protein-coding genes in shell-less octopods suggests that regulatory changes, rather than structural ones,^32^^,^^42^ facilitated shell degeneration. Fossil record and molecular data provide a temporal framework to time of these innovations (Figure 1A), including the accumulation of chromosomal rearrangements and their impacts on phenotypic evolution in the octopod lineage.

Taken together, our findings have important implications for understanding the directionality of chromosomal evolution in the animal kingdom, its long-term impact on the emergence of novel putative regulatory regions, and, eventually, morphological innovations. Further attempts to obtain a chromosomal-scale assembly of *Vampyroteuthis* will help to determine the exact karyotypic changes that occurred at the base of Octopodiformes.

### Limitations of the study

The biggest limitation of our study is the lack of *Vampyroteuthis* chromosomal-scale assembly. While several attempts were made to generate HiC library from the available material, we were not successful in the end. The use of simulated fragmented genomes and statistical analyses presented in this manuscript try to assess the confidence in our syntenic pattern estimation, which we found to be sufficient to propose the outlined scenario of karyotype fusions in octopods. While our study predicts larger number of chromosomes in *Vampyroteuthis* than in other octopodiform species, it does not rule out the possibility of fewer chromosomes. In this scenario, however, we still expect the *Vampyroteuthis* chromosomes to be comprised of independent and more recent fusions and generally retain at least parts of decapodiform-like synteny, with several fusion-with-mixing events clearly dating back to the octopod lineage. Importantly, our study should provide a contribution to a more thoughtful view of how changes to chromosomal identities and their homologies occur in cephalopods, highlighting the importance of irreversible fusion-with-mixing processes.

## Resource availability

### Lead contact

Further information and requests for resources and reagents should be directed to and will be fulfilled by the lead contact Masa-aki Yoshida (mayoshida@life.shimane-u.ac.jp).

### Materials availability

This study did not generate new unique reagents.

### Data and code availability

Genome and transcriptome sequencing reads were deposited in the Sequence Read Archive (BioProject IDs; *Vampyroteuthis* DNA, PRJDB14554; *Vampyroteuthis* RNA, PRJDB14554; *A. hians* DNA, PRJDB14523; *A. hians* RNA, PRJDB14523).

Genome assemblies were deposited in the DNA DataBank of Japan (INSDC) via the following accession numbers; Genbank: BAAFZU010000001-99 for *Vampyroteuthis*, and Ganbank: BAAFZT010000001-389 for *A. hians*. Other related files are available through the figshare collection; https://doi.org/10.6084/m9.figshare.c.7732304. Code and procedures are described in https://bitbucket.org/viemet/public/src/master/vamp/.

## Acknowledgments

We thank Noriyoshi Sato (10.13039/501100010655Tokai University) for providing *Vampyroteuthis* samples. We also thank all members of the Simakov lab (University of Vienna), in particular Thea Rogers and Darrin Schultz, for invaluable discussions and comments on the manuscript. D.H.E.S. and K.H. would like to thank Nanami Tochino (Setiamarga Lab at NIT Wakayama), for her assistance on the phylogeny/timetree inference. We thank three anonymous reviewers for their valuable input on this manuscript.

Genome sequencings of *Vampyroteuthis* sp. and *A*. *hians* were supported by 10.13039/501100001691JSPS KAKENHI grant no JP22H04925 (Platform for Advanced Genome Science). M.A.Y. was partially supported by Takeda Science Foundation Life Science Research grants FY 2023, and KAKENHI Grants-in-aid for Basic Research (no. 22K06340) awarded to M.A.Y. and D.H.E.S. M.A.Y. also thank the Faculty of Life and Environmental Sciences at 10.13039/501100004318Shimane University for the financial support for publishing this report. D.H.E.S. was partially supported by KAKENHI Grants-in-aid for Basic Research (19K12424 and 23K11511), Takeda Science Foundation for Life Sciences Research grant FY 2022, and the National Institute of Technology GEAR 5.0 Project for Agriculture, Forestry, and Fisheries to support K.H.’s position in Setiamarga lab. K.H. was partially supported by Sasakawa Scientific Research grant FY 2024 from the Japan Science Society and JST SPRING GX grant (no. 23A360), both of which are fellowships for graduate students. O.S. was supported by the European Research Council’s Horizon 2020: European Union Research and Innovation Programme, grant no. 945026. The computational results of this work have been achieved using the Life Science Compute Cluster (LiSC) of the University of Vienna.

## Author contributions

This work is a result of an equal collaboration among three labs led by three principal investigators (the corresponding authors: M.A.Y., D.H.E.S., and O.S.), all giving their shares equally toward the completion of this project. Conceptualization, funding acquisition, project administration, M.A.Y., D.H.E.S. and O.S.; investigation, formal analysis, A.T., H.T., H.N., E.T., K.K.-N, T.K., K.H., H.M., M.T., and H.N.; resources, A.T., H.T., and H.N.; writing – original draft; M.A.Y., D.H.E.S., and O.S. All authors contributed to the final manuscript.

## Declaration of interests

The authors declare no competing interests.

## STAR★Methods

### Key resources table

**Table:** 

REAGENT or RESOURCE	SOURCE	IDENTIFIER
**Biological samples**		

1 *Vampyroteuthis* individual	Research cruise bycatch	GenBank: SAMD00553067
1 *A. hians* individual	Fishery bycatch	GenBank: SAMD00553068

**Deposited data**		

Short and long read raw data for *Vampyroteuthis*	This paper	GenBank: PRJDB14554
*Vampyroteuthis* genome assembly (ver 1.0)	This paper	GenBank: BAAFZU010000001-BAAFZU010012099
*Vampyroteuthis* genome assembly (ver 2.0)	This paper	Figshare: https://doi.org/10.6084/m9.figshare.28639172
Short and long read raw data for *Argonauta*	This paper	GenBank: PRJDB14523
*Argonauta* genome assembly (ver 1.0)	This paper	GenBank: BAAFZT010000001-BAAFZT010000389
*Argonauta* genome assembly (ver 2.0)	This paper	Figshare: https://doi.org/10.6084/m9.figshare.28639166
*Doryteuthis pealeii* genome	Genbank	GCA_023376005.1
*Euprymna scolopes* genome	Genbank	GCA_024364805.1
*Octopus vulgaris* genome	Genbank	GCA_951406725.2
*Sepia lycidas* genome	Genbank	GCA_963932145.1
*Eledone cirrhosa* genome	Genbank	GCA_964016885.1
*Thysanoteuthis rhombus* genome	Genbank	GCA_963457665.1
*Nautilus pompilius* genome	Genbank	GCA_047652355.1

**Software and algorithms**		

Jellyfish 2.2.10	Marçais and Kingsford 2011^43^	–
Genomescope2 v2.0	Ranallo-Benavidez et al. 2020^44^	–
DeepConsensus v1.1.0	Baid et al. 2023^45^	–
Hifiasm 0.19.3-r572	Cheng et al. 2021^46^, 2022^47^	–
Python3.7	van Rossum and de Boer^48^	–
RepeatModeler 2.0.5	Flynn et al. 2020^49^	–
RECON 1.08	Bao and Eddy 2002^50^	–
RepeatScout 1.0.6	Price et al. 2005^51^	–
RMBlast 2.9.0	http://www.repeatmasker.org/RMBlast.html	–
LtrHarvest 1.5.9	Ellinghaus 2008^52^	–
Ltr_retriever 2.6	Ou and Jiang 2018^53^	–
Ltrdetector 1.0	Valencia and Girgis 2019^54^	–
RepeatMasker 4.1.5	Smit et al. 2013^55^	–
Minimap2 2.28-r1221-dirty	Li 2018^56^	–
Purge_dups 1.2.5	Guan et al. 2020^57^	–
GINGER 1.0.1	Taniguchi et al. 2024^58^	–
nextflow 21.10.0	Tommaso et al. 2017^59^	–
Juicer v1.6	Juicer v1.6^60^	–
3D-DNA v180419	3D-DNA v180419^61^	–
Juicebox v1.11.08	Juicebox v1.11.08^62^	–
custom R (version 4.2.2) script psyntPlot.R	This paper	https://bitbucket.org/viemet/public/src/master/vamp/
MAFFT v7	Katoh et al. 2019^63^	–
Gblocks v0.91b	Castresana 2000^64^	–
MEGA11	Tamura et al. 2021^65^	–
PartitionFinder v2.1.1	Lanfear et al. 2017^66^	–
RAxML-HPC v7.2.8	Stamatakis 2014^67^^,^^68^	–
PAML v4.0	Yang 2007^69^	–
MCMCTREE	Inoue et al. 2010^70^	–
miniprot	Li 2023^71^	–

### Experimental model and study participant details

*Vampyroteuthis* sp. used for sequencing was derived from a single individual (juvenile, unknown sex) caught in Suruga bay by T/V Hokuto of Tokai University on the voyage of 2022-06-07. The *A. hians* used for sequencing was derived from a single, matured female caught as a bycatch in the fixed nets set along the coast in Oki Island, Shimane Prefecture, Japan (36°17′20.6″ ″N 133°12′46.4″″E) on 2021-10-04. Since both animals were collected from wild populations, their exact ages could not be determined. The egg-shell of the *A. hians* was deposited in the University Museum, the University of Tokyo with voucher ID: UMUT RM34224.

### Method details

#### DATA generation

Whole-genome shotgun sequencing was performed using PacBio and Illumina sequencing platforms. Genomic DNA was extracted from the mantle tissue of *Vampyroteuthis* sp. and the ovary of *A. hians* using a Genomic-tip Kit (QIAGEN, Hilden, Germany) and was sheared into fragment sizes ranging from 15 kb to 20 kb with a g-tube device (Covaris Inc., MA, USA). PacBio HiFi libraries for *Vampyroteuthis* sp. and *A. hians* were prepared using a SMRTbell Prep Kit 3.0 (Pacific Bioscience, CA, USA) according to the manufacturer’s instructions and were size-selected using the SageELF system (Saga Science, MA, USA). Seventeen SMRT cells for *Vampyroteuthis* libraries and two SMRT cells for *A. hians* libraries were sequenced on the PacBio Sequel II/IIe systems with Binding Kit 3.2 and Sequencing Kit 2.0 (30 hours collection times). The consensus (HiFi) reads were generated from raw full-pass subreads using the DeepConsensus v1.1.0 program^45^ with the default parameter settings.

For Illumina sequencing, genomic DNA was fragmented to an average size of 500 bp using the Focused-ultrasonicator M220 (Covaris Inc., MA. USA). Paired-end libraries were constructed with a TruSeq DNA PCR-Free Library Prep kit (Illumina, CA, USA) and size-selected on an agarose gel with a Zymoclean Large Fragment DNA Recovery Kit (Zymo Research, CA. USA). The final libraries were sequenced on the NovaSeq 6000 system (Illumina, San Diego, CA, USA) with 2 × 150 bp read length.

Total RNA was extracted from three *Vampyroteuthis tissues* (brain, beak, and eyes) and four *A. hians* tissues (brain, eyes, heart, and embryo) using an E.Z.N.A. Mollusc RNA kit (Omega Bio-Tek, GA. USA) and a Nucleospin RNA clean-up XS (TaKaRa, Japan). RNA libraries were constructed using an Illumina Stranded mRNA Prep, Ligation (Illumina, San Diego, CA, USA) following the manufacturer’s instructions. Sequencing was conducted on the NovaSeq 6000 system with 2 x 100 bp read length. The concentration and quality of the libraries were assessed using the Qubit 4 Fluorometer (Thermo Fisher Scientific, MA, USA), the 2100 Bioanalyzer system (Agilent Technologies, CA, USA), and the 7900HT Fast Real-Time PCR System (Thermo Fisher Scientific, MA, USA).

### Quantification and statistical analysis

#### Whole genome assembly and annotation

The PacBio libraries were constructed using the genomic DNA from the *Vampyroteuthis* sample. In total, 571,504,779,822 bp of HiFi reads were obtained by PacBio Sequel II/IIe. The total sequencing depth was approximately 40X, and the average read length was 16.3 kb. Short reads by illumina NovaSeq6000 (PE500) were also obtained, and 1,303,449,174,900 bp of clean data were acquired. Three RNA-seq libraries were obtained from the same individuals of *Vampyroteuthis*.

We assembled the whole genome by Hifiasm^46^^,^^47^ v.0.19.3-r572 with an option --hg-size 14g since preliminary k-mer analysis showed a genome size of 14 Gb (Table S1). We note that the results of the vampire squid stably reproduced 14G assembly size regardless of options. The v1.0 assembly we showed has a genome size of 14.7 Gbp and an N50 length of 7.6 Mbp. The genome size was 14.5 Gbp and N50 length 7.8 Mbp as a result of option 12G specification (hifiasm --hg-size 12g). Even using the latest version of hifiasm, the genome size was 14.4 Gbp and N50 length 8.5 Mbp with the 12G designation, and the N50 was extended a little further (hifiasm-0.24.0-r702 --hg-size 12g, 2024Dec_ver). Thus, regardless of the genome size setting option, the final assembly size remains the same across the currently available methods. BUSCO analysis showed that its completeness was as high as 97.2. This score was comparable to other chromosome-level Molluscan genomes (Table S2).

K-mer based genome size estimation was applied by GenomeScope2.0^44^ (-k 35 -p 2). Jellyfish^43^ run was performed with an option: high 10000000. The estimated genome size was 11.5 Gb, and heterozygosity was estimated to be 1.8-2% (Figure S1). Another k-mer condition (k=27) gave almost the same result (11.8 Gb). The Hifiasm standard output size is significantly larger than the genome size estimated by k-mer analysis of short-read data. The discrepancy in assembly size is probably the result of duplication due to haplotype diversity. BUSCO analysis also detected a high number of duplicate genes (Table S3). Based on these findings, contig duplication was suspected. We applied purge_dups to reduce the duplicated content to a haplotype consensus sequence. The purged assembly data (v2.0) set has a size of 11.7 Gb and 5,616 contigs with an N50 contig size of 9.96 Mbp. GenomeScope 2.0 estimated an expected genome size of 11.5-11.8 Gb, which is close to the assembled size after applying purge_dups. The purge_dups collapses the haplotype-separated assembly and reduces the duplicated content to a haplotype consensus sequence, therefore, contigs purged in this process are probably duplicated contigs due to haplotype diversity. The purged contigs are not enriched with a high mask rate and appear to be a population of contigs derived from minor haplotigs with the same distribution as the whole (Figure S1). This is also in good agreement with the improved duplicate comparing BUSCO scores before and after purge_dups (7.1% to 3.5%). For *Argonauta hians*, GenomeScope 2.0 estimated an expected genome size of 1.56 Gbp, which is consistent with the assembled genome size (1.63 Gbp; version 1.0). Repetitive elements of the *Vampyroteuthis* genome were estimated with Repeatmodeler.^49^ However, LTR structural analysis (LtrHarvest::gt command) was not finished for over a month, probably due to the large-sized genome. So, we applied an alternative method; the LTRs that were not detected by repeatmodeler (-LTRStruct off), and additionally estimated using ltrDetector.^54^ The query was compared to the classified sequences in “rpmd_db2-families.fa” using RepeatMasker version 4.1.2-p1 (default mode) with rmblastn version 2.11.0+. This method is practical and finished within a day. 62.47% of the genome was estimated as repetitive regions and masked for the following analysis (Table S4). For *A. hians*, a repeat mask was followed by an estimation of the gene model. The repeat libraries were generated using RepeatModeler v2.0.5^49^ with RECON v1.08,^50^ RepeatScout v1.0.6,^51^ and RMBlast v2.9.0 (http://www.repeatmasker.org/RMBlast.html). Long terminal repeat sequences were also identified with “-LTRStruct” option, using LtrHarvest v1.5.9^51^ and Ltr_retriever v2.6.^53^ Repeat annotation was performed with RepeatMasker v4.1.5^55^ using the custom libraries.

Gene prediction models were generated using GINGER 1.0.1.^58^ In brief, this pipeline combines *ab initio*, RNA-seq-based, and homology-based prediction results for related species. For homology-based prediction, the amino acid sequences of *Octopus vulgaris*,^72^ *O. bimaculoides*,^14^ *Architeuthis dux*,^73^ *Crassostrea gigas*,^74^ and *Mizuhopecten yessoensis*^75^ were utilized and mapped to the genome scaffolds. The predicted genes were evaluated using BUSCO,^76^ and resulted in 96.6% complete genes being marked, suggesting high accuracy of the annotation [C:96.6%[S:93.9%,D:2.7%],F:1.9%,M:1.5%,n:954]. The initial gene prediction using Ginger yielded a total of 82,025 gene models. This number is extremely high compared to other octopus species, likely due to the difficulty of accurately masking repeat sequences in large genomes. To address this, we applied a series of filtering steps to remove spurious gene predictions. Specifically, we excluded genes overlapping with LTRs identified by ltrDetector but not masked by RepeatMasker, and we removed genes lacking homology to related species, identifiable protein domains, or RNA-seq support. Although these filters significantly reduced the total gene count, the BUSCO completeness score remained unchanged. The final gene set for the *Vampyroteuthis* genome includes 62,225 protein-coding genes.

For *A. hians*, PacBio library was constructed using the genomic DNA from a single individual. In total, 94,777,318,231 bp of HiFi reads were generated by PacBio Sequel II/IIe. In addition, short-read sequencing was performed on the same sample using the Illumina NovaSeq 6000 platform (PE500), yielding 167,870,168,100 bp of clean data. of clean data. Four RNA-seq libraries were obtained from the same individuals of *A. hians*. *De novo* genome assembly was conducted using Hifiasm with default parameters, resulting in an initial assembly of 389 contigs with an N50 of 30.1 Mb and a maximum contig length of 138.0 Mb. Hi-C libraries for scaffolding were constructed using Dovetail Omni-C Kits (Cantata Bio, CA, USA), and were sequenced on an Illumina MiSeq system. The Hi-C reads were mapped to the contigs and filtered using Juicer v1.6.^60^ Scaffolding was performed with 3D-DNA v180419.^61^ The resulting scaffolds were manually inspected and refined using Juicebox v1.11.08.^62^ The scaffolding successfully performed to give rise to chromosomal level. *A. hians* genome (ver2) was further assembled into 28 scaffolds with an N50 of 60.9 Mb and a maximum scaffold length of 223.2 Mb at the chromosomal level. For *A. hians*, a repeat mask was followed by an estimation of the gene model. The repeat libraries were generated using RepeatModeler v2.0.5^49^ with RECON v1.08,^50^ RepeatScout v1.0.6,^51^ and RMBlast v2.9.0 (http://www.repeatmasker.org/RMBlast.html). Long terminal repeat sequences were also identified with “-LTRStruct” option, using LtrHarvest v1.5.9^52^ and Ltr_retriever v2.6.^53^ Repeat annotation was performed with RepeatMasker v4.1.5^55^ using the custom libraries. Genome annotation was conducted with GINGER v1.0.1.^58^ The conda environment was created with Python v3.7, and nextflow v21.10.0^59^ was installed using conda. In the homology search of GINGER, the protein database consists of amino acid sequences from six mollusks, *Argonauta argo*,^32^ *Octopus vulgaris*,^72^ *O. bimaculoides*,^14^ *Architeuthis dux*,^73^ *Crassostrea gigas*,^74^ and *Mizuhopecten yessoensis*,^75^ and the parameter files of Spaln are all ‘InsectDB’.

#### Phylogenetic inference

The phylogenetic tree of cephalopod lineages (Figure 1A) was inferred using mitochondrial genomes (13 protein-coding genes [PCGs], two rRNA-coding genes, and 22 tRNA-coding genes) and nuclear ribosomal RNA-coding genes (18S-rRNA and 28S-rRNA), totaling 39 genes. First, the nucleotide sequences of each gene were aligned using the online version of MAFFT v7.^63^ For PCGs, amino acid sequences were used as alignment constraints. The Q-INS-i algorithm was applied for nucleotide alignments of rRNAs and tRNAs, while the E-INS-i algorithm was used for amino acid alignments of PCGs. Post-alignment sequence editing was performed using Gblocks v0.91b with the least stringent settings, with the codon option applied for PCGs.^64^ AIC- and BIC-based model selection was conducted in MEGA11.^65^ PartitionFinder v2.1.1^66^ was used to determine the optimal partitioning scheme for each concatenated dataset, resulting in 40 partitions. The maximum likelihood tree was inferred using RAxML-HPC v7.2.8^67^^,^^68^ under the GTR+G+I model, with 1,000 bootstrap replicates under the rapid bootstrap algorithm.

#### Divergence time estimation

The resulting maximum likelihood tree topology and the concatenated dataset of 39 genes, divided into 40 partitions, were used for partitioned substitution rate inference in PAML v4.0.^69^ Divergence time estimation was conducted using MCMCTREE.^70^

Five fossil calibration points were used: (1) crown Mollusca, upper-constrained to 549 Ma based on Cloudinids^77^; (2) Nautiloidea–Coleoidea divergence (crown Cephalopoda), lower-constrained to ∼408 Ma (Lower Devonian)^78^; (3) Argonauta vs. Octopus split, lower-constrained to ∼29 Ma (Early Oligocene)^79^; (4) Decapodiformes–Octopodiformes split (crown Coleoidea), lower-constrained to 240 Ma (Middle Triassic Germanoteuthis)^78^^,^^80^; and (5) Vampyromorpha–Octopoda split (crown Octopodiformes), lower-constrained to 162 Ma based on Loligosepia (Early Jurassic).^81^ Fossil calibration information was listed in the Table S5.

#### Orthology and syntenic analysis

For consistency in orthology handling, we mapped *Doryteuthis pealeii* and *Octopus vulgaris* peptides to all genomes using miniprot^71^ 0.12 and using -gff option as an output. In total, 30432 *D. pealeii* and 25682 *O. vulgaris* genes were mapped. Custom scripts were used to parse the mapping results and convert them into pairwise synteny files (.psynt) for each species pair (available on the repository link below). Each .psynt file contains information on mutual best orthologs as well as their genomic locations in two species. Only contigs with 15 or more orthologous genes were used. Dotplots were made using a custom R (version 4.2.2) script psyntPlot.R using published procedures (https://bitbucket.org/viemet/public/src/master/vamp/). Significance of chromosomal homologies was tested using Fisher’s exact test, and associations of adjusted p-value (Bonferroni correction) of 0.05 are shown as black colored dots. Clustering of contigs or chromosomes on dotplots was done either by predefined order of chromosomes (Figure 2) to enable multi-species comparisons, or via Euclidean distance measure on the number of shared orthologs and ward.D2 clustering in R. Statistical analysis of mixing was done with TraMineR 2.2-11 package in R.^82^

Whole genome alignments were done on hard-masked genomes (RepeatModeler 2.0.6 and RepeatMasker 4.1.8) with blastn 2.16.0+ using the following parameters: -task megablast -perc_identity 0 -template_length 16 -penalty -2 -word_size 11 -evalue 1 -template_type coding_and_optimal.^83^ Transdecoder 5.7.1 was used to predict open reading frames on conserved aligned sequences (TransDecoder.LongOrfs -m10 parameter was used) and classify alignments into coding vs non-coding regions, in addition to available gene annotation overlap. Only alignments of 50 or more base pairs were reported. All code, data files, and protocols are available under https://bitbucket.org/viemet/public/src/master/vamp/. All dotplots for every figure and their supporting data can be generated by running the prepInput.sh and plot_figures.R scripts on the repository.

## Supplemental information

Document S1. Figures S1–S8 and Tables S1–S5

Data S1. Mixing data statistics for normalized turbulence analysis
