# Supplementary material for: Giant genome of the vampire squid reveals the derived state of modern octopod karyotypes
Source: iScience. 2025 Oct 23;28(11):113832. doi: 10.1016/j.isci.2025.113832 (PMC12682272; doi:10.1016/j.isci.2025.113832)
Supplement: Document S1. Figures S1–S8 and Tables S1–S5 [file mmc1.pdf]

iScience, Volume *28*

Supplemental information

Giant genome of the vampire squid reveals the derived state of modern octopod karyotypes

Masa-aki Yoshida, Emese Tóth, Koto Kon-Nanjo, Tetsuo Kon, Kazuki Hirota, Atsushi Toyoda, Hidehiro Toh, Hideyuki Miyazawa, Makoto Terauchi, Hideki Noguchi, Davin H. E. Setiamarga, and Oleg Simakov

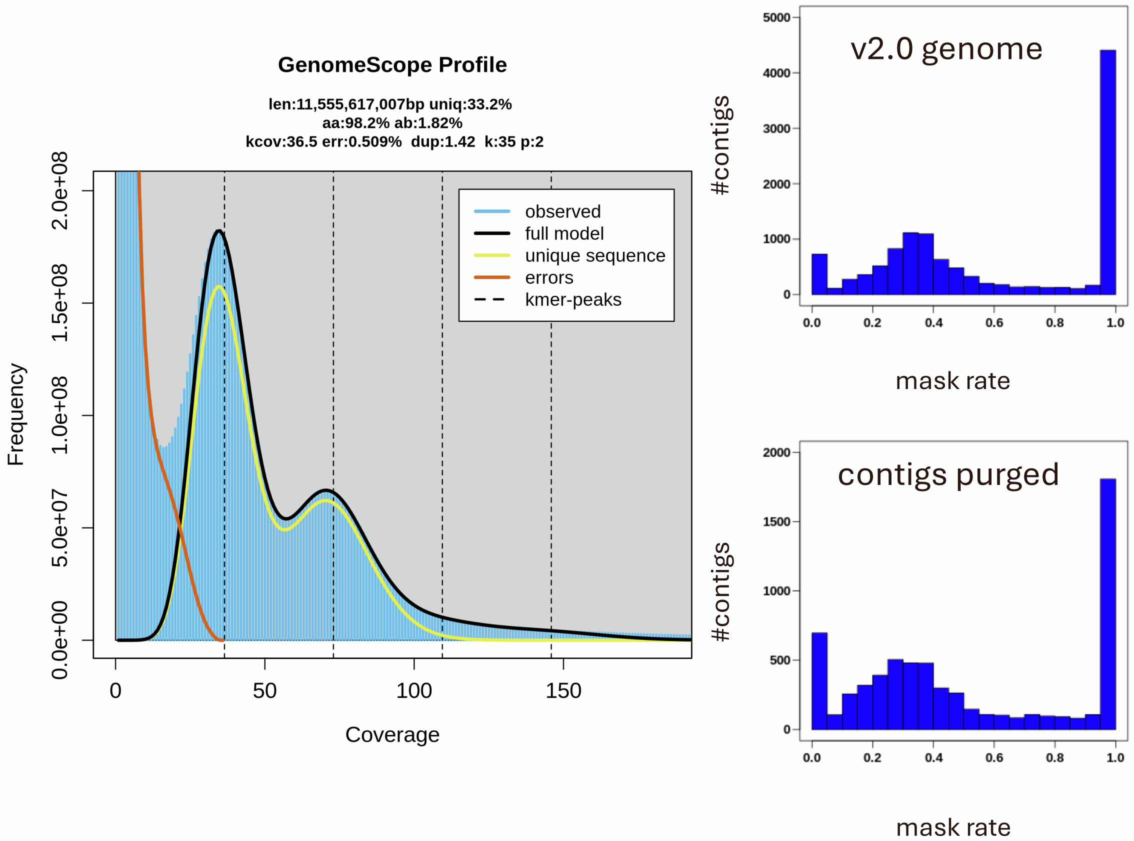

Supplementary Figure 1. **Genome size estimation via GenomeScope2.0.** Estimated results at k=35 are shown; estimates at k=27 were almost identical. High heterozygosity rate (1.82%) indicates the possible existence of overlapping contigs due to high heterozygosity. The assembly size of the ver 2.0 genome is close to the estimated genome size. The purged contig is not enriched with a high mask rate. There is a possibility that the current assembly contains duplicated contigs.

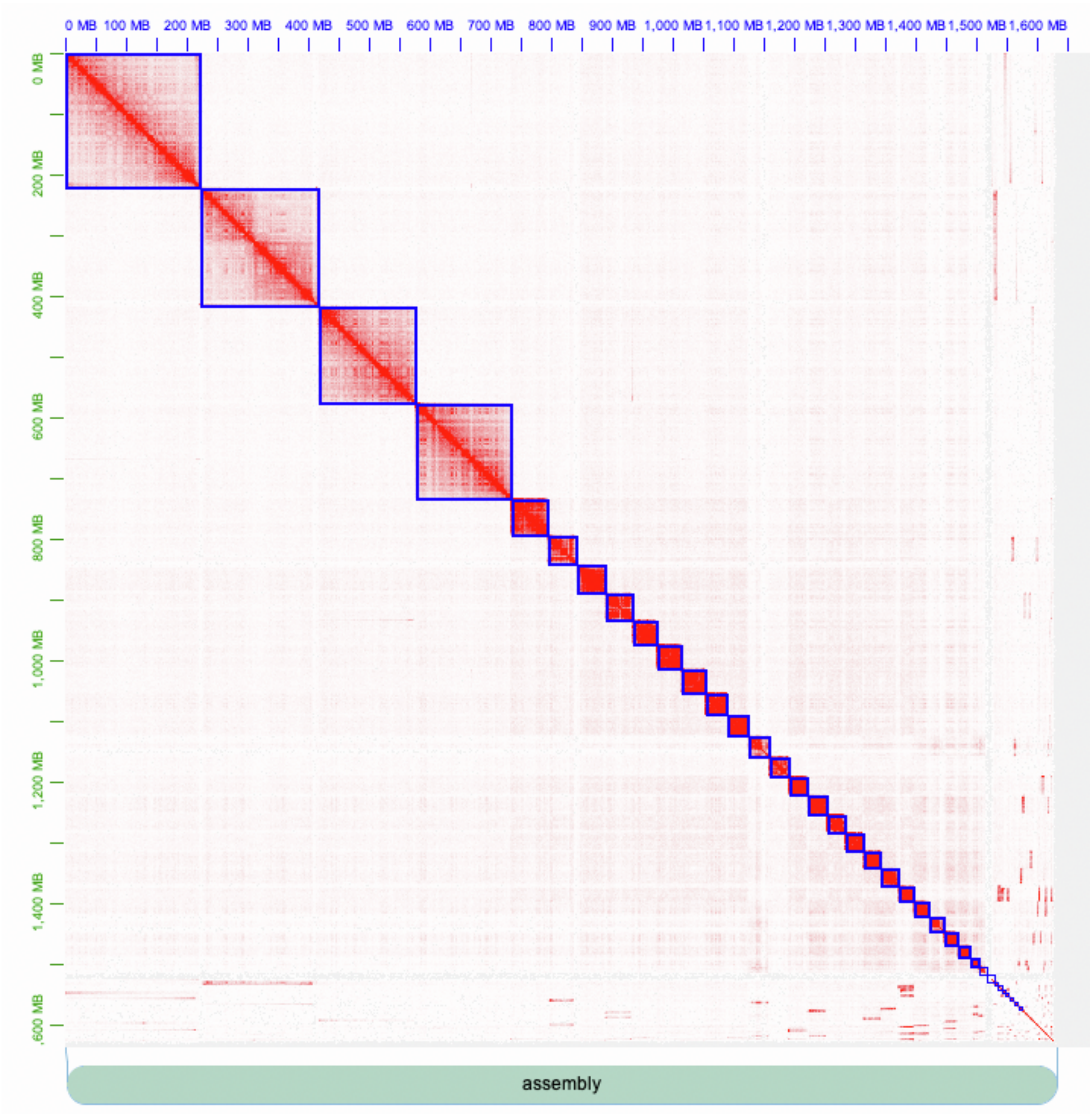

Supplementary Figure 2. ***Argonauta hians* HiC map**.

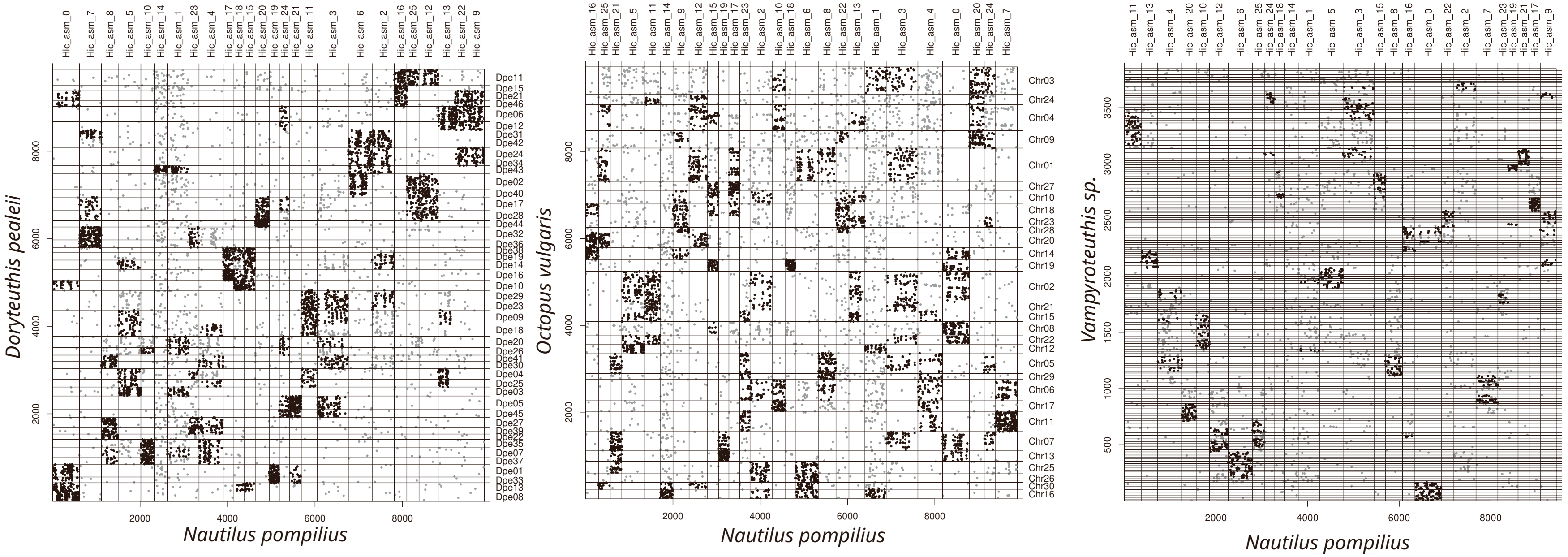

Supplementary Figure 3. **Translocation-rich history in coleoid genomes.** *Octopus vulgaris* and *Doryteuthis pealeii* genomes show many inter-chromosomal translocations and fusion-with-mixings compared to the Nautilus genome, which represents the ancestral molluscan karyotype. Significant associations (Fisher's exact test p-value <0.05) are labelled in bold color.

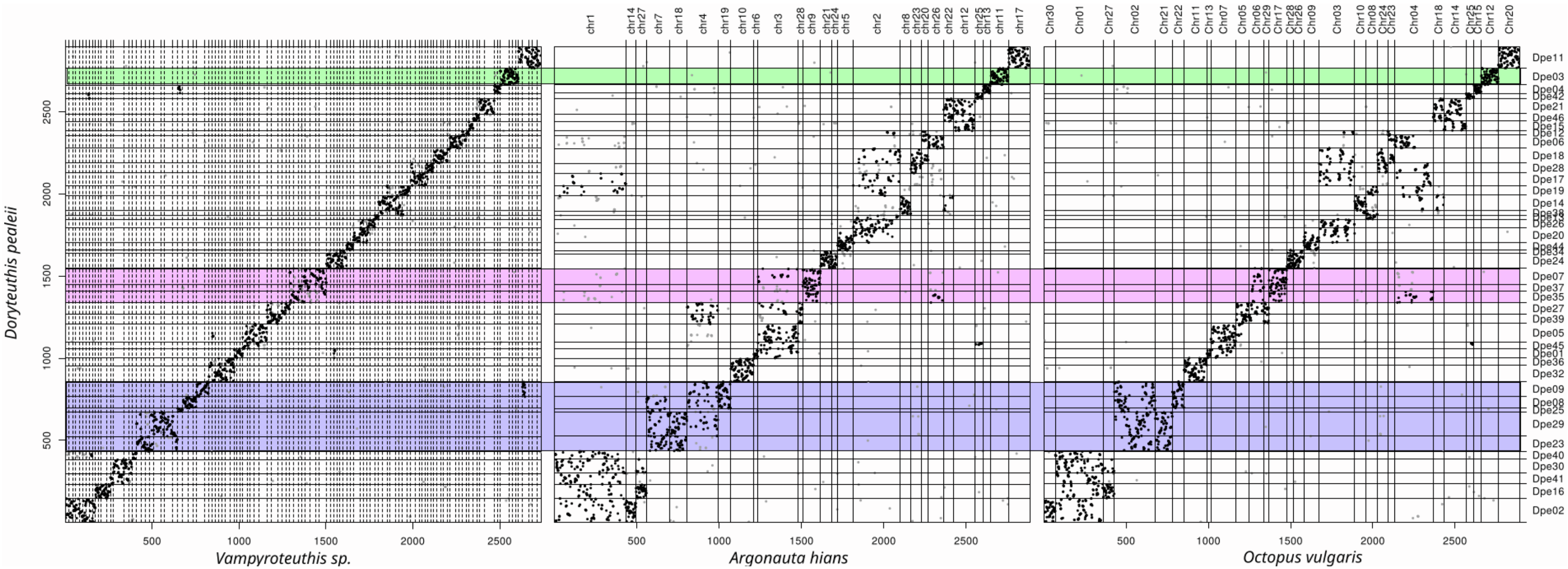

Supplementary Figure 4. **Syntenic pattern using a different orthology set (*O. vulgaris* gene annotations as reference)**. Same arrangements as in Figure 2. The color codings indicate representative examples of the ancestral coleoid chromosome (green), ancestral Octopodiformes mixed chromosome (pink), and fused-and-mixed chromosomes in octopods (blue), respectively.

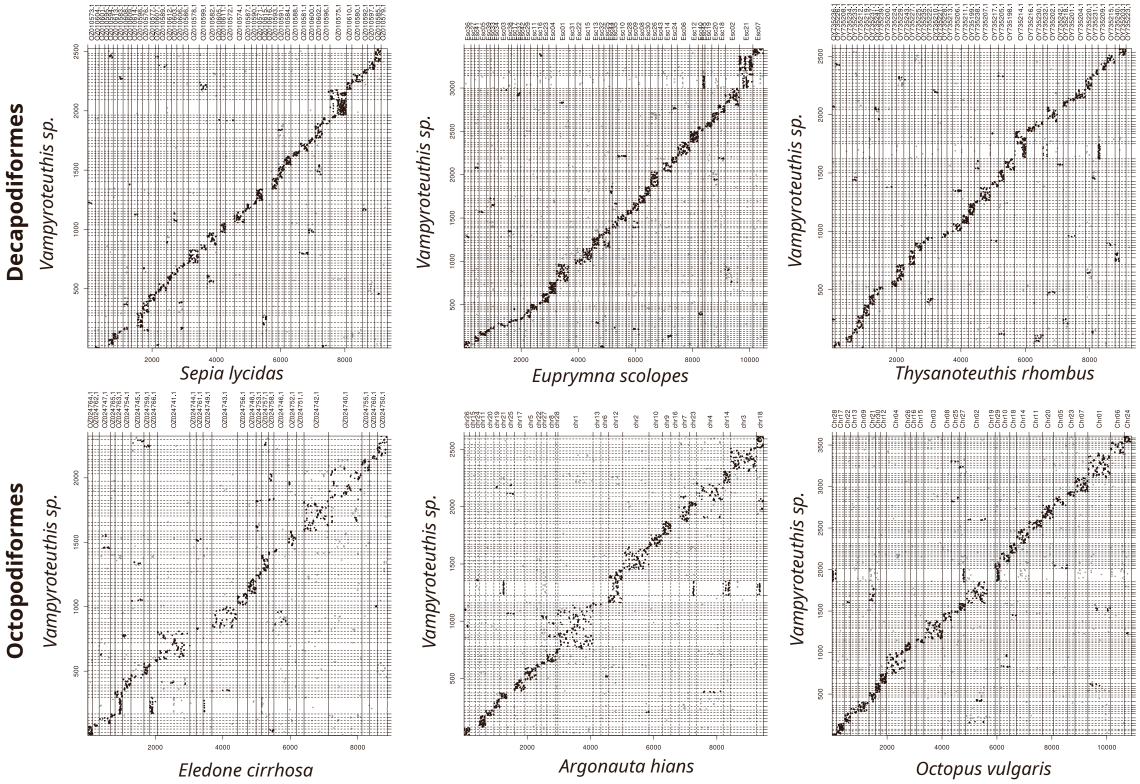

Supplementary Figure 5. ***Vampyroteuthis* shows an intermediate karyotype between Octopodiformes and Decapodiformes.** Significant associations (Fisher's exact test p-value <0.05) are labeled in bold color.

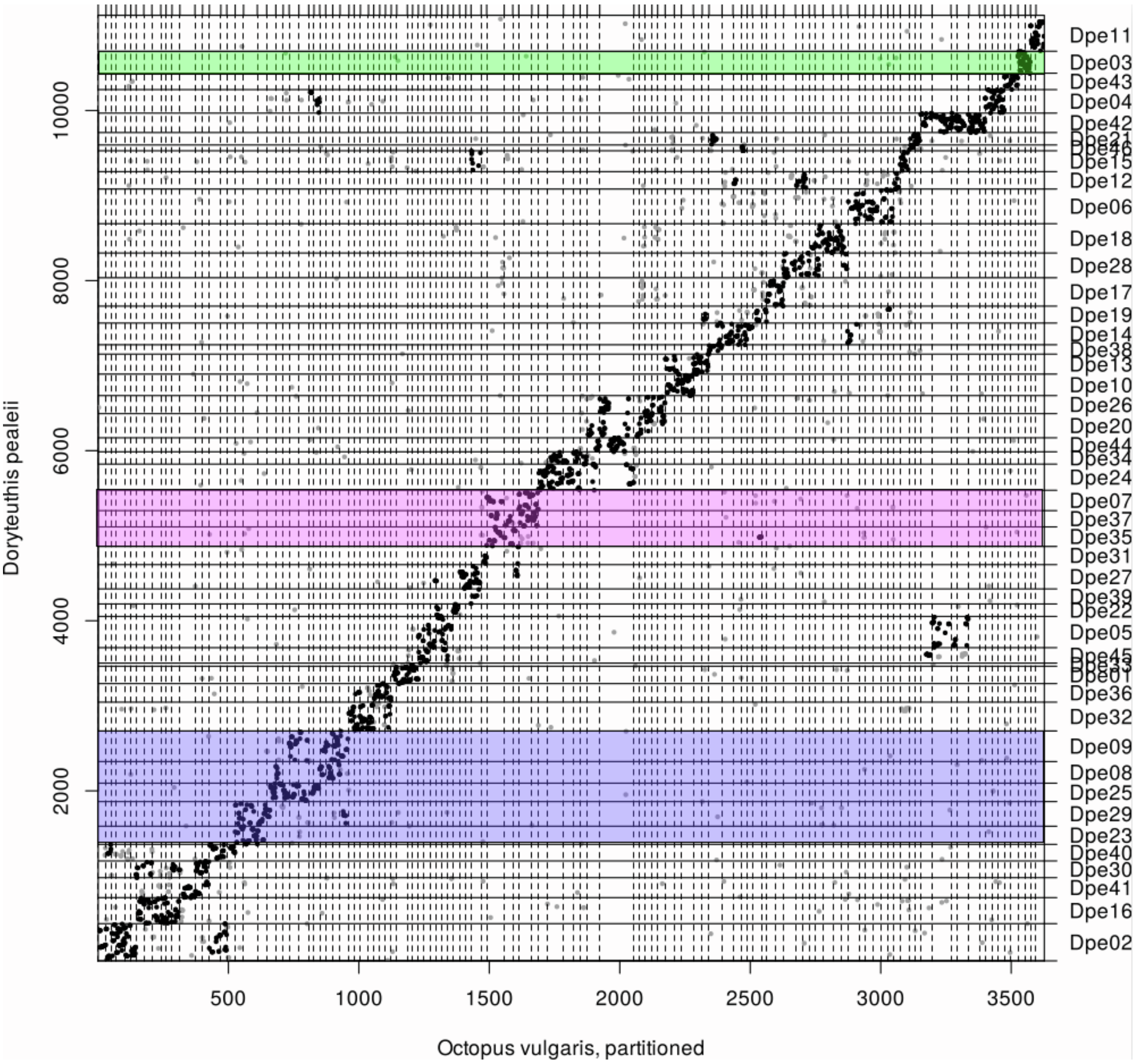

Supplementary Figure 6. ***Octopus vulgaris* genome, broken up into pieces of similar size as *Vampyroteuthis* contigs, recapitulates FWMs seen with chromosomal-level assembly.** Significant associations (Fisher's exact test p-value <0.05) are labeled in bold color. The color codings indicate representative examples of the ancestral coleoid chromosome (green), ancestral Octopodiformes mixed chromosome (pink), and fused-and-mixed chromosomes in octopods (blue), respectively.

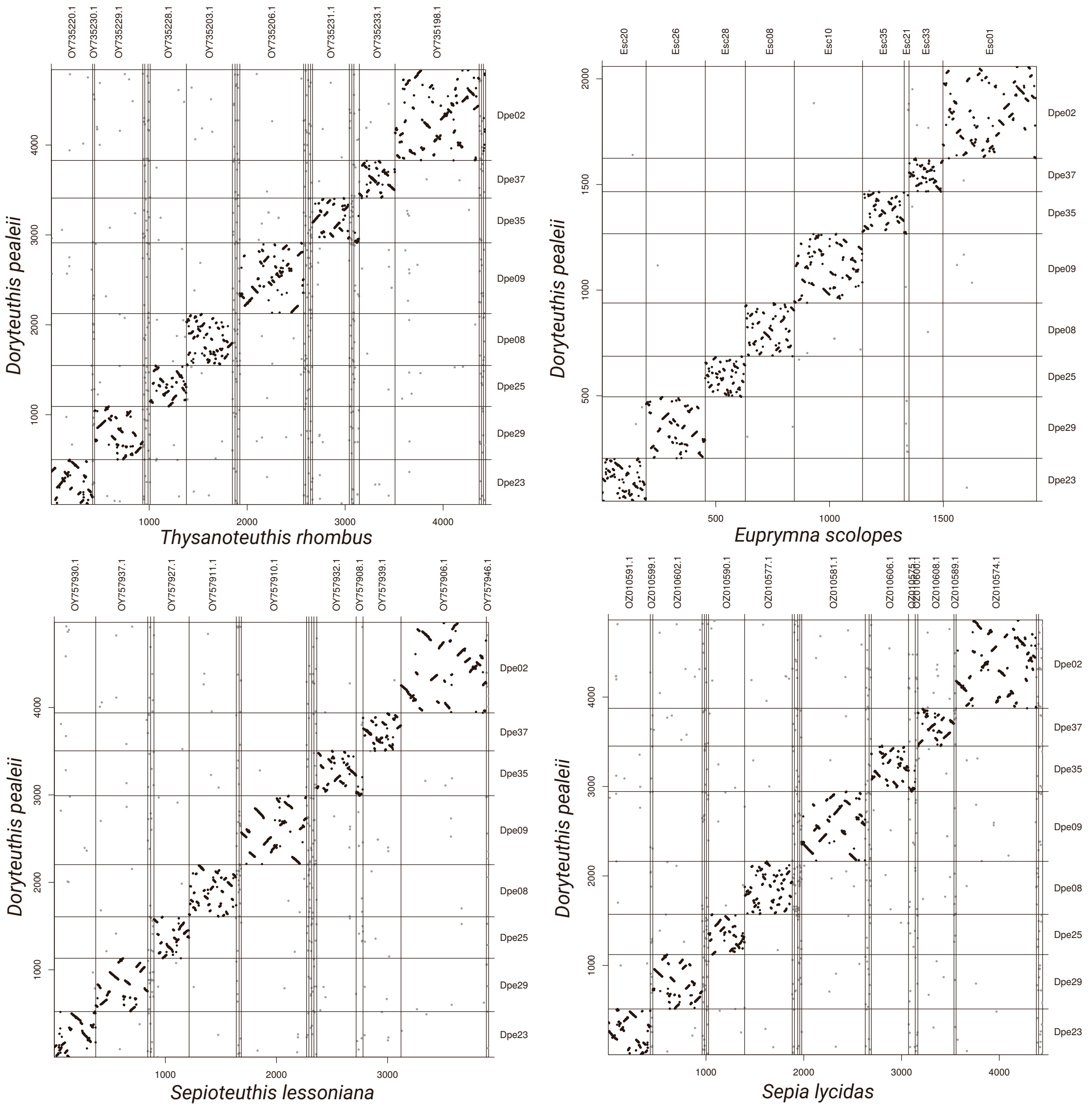

Supplementary Figure 7. **Decapodiformes chromosomes are conserved across their major lineages.** Dotplots for the selected (Figure 2) *Doryteuthis* chromosomes are shown, highlighting the absence of any "cryptic" chromosomal homologies (all chromosomes show 1-1 homology) in comparison with pelagic squids, as well as a bobtail squid and a cuttlefish. Significant associations (Fisher's exact test p-value <0.05) are labeled in bold color.

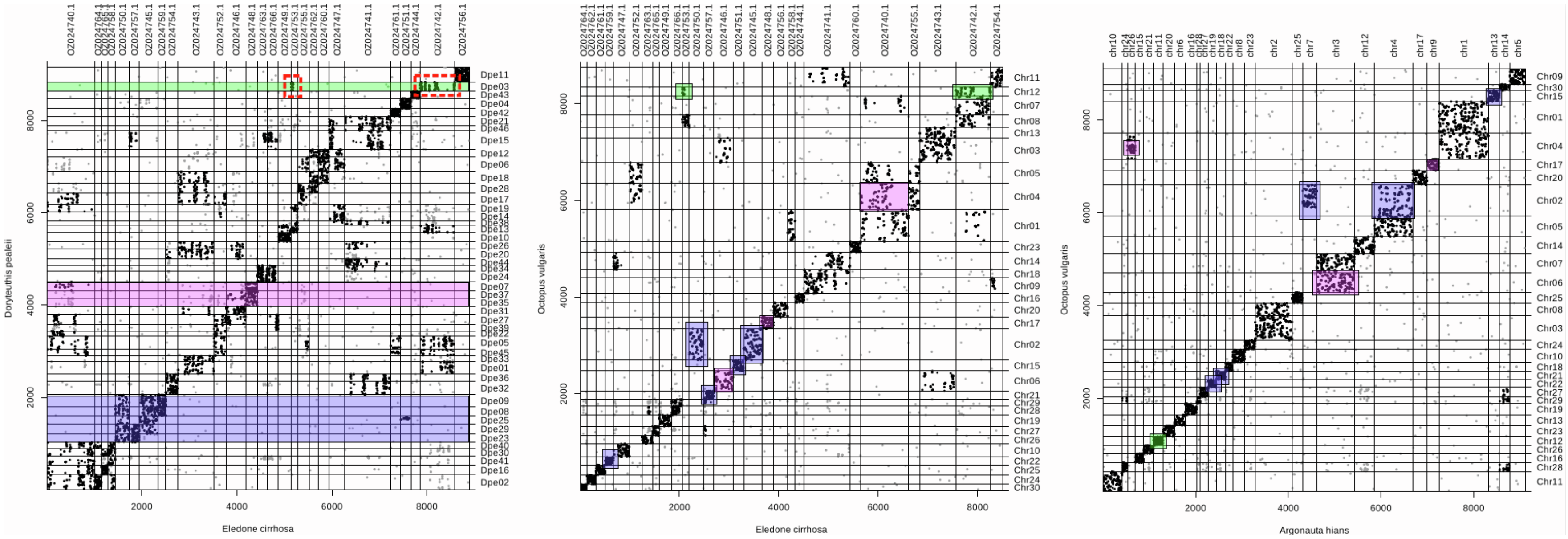

Supplementary Figure 8. ***Eledone* shows a more derived karyotype**. Dotplots within octopod genomes highlight high conservation between *A. hians* and *O. vulgaris*. *Eledone*, on the other hand, has additional translocations with one ancestral coleoid unit (Dpe03-Ovu12) split across multiple chromosomes. The color codings indicate representative examples of the ancestral coleoid chromosome (green), ancestral Octopodiformes mixed chromosome (pink), and fused-and-mixed chromosomes in octopods (blue), respectively. Red dashed boxes show chromosome that is split in *Eledone* but occurs as single unit in other octopods.

Supplemental Table 1. Statistical comparison genome assemblies shown in this paper. Two Vampyroteuthis assemblies were generated with v1 being the intermediate state and v2 our working draft of the genome. Similarly, for Argonauta, v1 being the intermediate state and v2 our working draft of the genome. The possibility that some genetic information has been lost from the contig information included in version 1 cannot be ruled out, and both are left to be reported.

Genome version	**Vampyroteuthis sp. v1.0**	**Vampyroteuthis sp. v2.0**	**A. hians v1.0**	**A. hians v2.0**
Difference from other versions	hifiasm standard output	duplicates purged using purge_dups	hifiasm standard output	Hi-C scaffolding
Total nucleotides	14,690,897,198	11,710,348,364	1,636,068,110	1,517,914,136
Number of contigs	12,099	5,616	389	28
N50	7,566,590	9,963,774	30,166,387	60,915,548
L50	490	326	18	5
Longest contig	51,721,974	51,721,974	138,080,145	223,252,000
Average length	1,214,224.08	2,085,175.99	4,205,830.62	54,211,219.14

Supplemental Table 2. Statistical comparison of cephalopod genomes

	genome size	n_proteins	busco proteins mode (metazoa_odb10, n:954)
Vampyroteuthis infernalis (v1.0)	14.6 gb	88,329	C:97.2%[S:91.2%,D:6.0%],F:2.0%,M:0.8%
Argonauta argo	1.3 gb	20,293	C:95.4%[S:93.1%,D:2.3%],F:2.6%,M:2.0%
Architeuthis dux	2.7 gb	51,225	C:85.5%[S:84.8%,D:0.7%],F:7.5%,M:7.0%
Crassostrea gigas	557 mb	51,045	C:95.4%[S:70.8%,D:24.6%],F:0.3%,M:4.3%
Mizuhopecten yessoensis	971 mb	41,567	C:98.6%[S:75.2%,D:23.4%],F:0.4%,M:1.0%
Octopus bimaculoides	2.3 gb	29,037	C:95.2%[S:69.8%,D:25.4%],F:2.3%,M:2.5%
Octopus vulgaris	2.7 gb	30,134	C:91.2%[S:67.2%,D:24.0%],F:3.7%,M:5.1%

Supplemental Table 3. BUSCO statistics for *Vampyroteuthis* genome assemblies showed the elimination of duplicated contigs by the purge_dups

	**Original assembly**	**After purge_dups applied**
version	1.0	2.0
genome size	14.6 Gb	11.7 Gb
N_contigs	12,095	5,616
repeats	60.8%	60.3%
busco (genome mode, vs metazoa_odb10, n:954)	C:95.8% [S:88.7%, D:7.1%], F:3.1%, M:1.1%	C:95.3% [S:91.8%, D:3.5%], F:3.2%, M:1.5%

Supplemental Table 4. Repeat content of the *Vampyroteuthis* genome (v.2.0) based on Repeat Masker output

		number of elements	Length occupied (bp)	Percentage
Retroelements		2,914,604	1,979,850,998	16.91 %
SINEs:		83,745	9217235	0.08%
Penelope		563,170	300,260,284	2.56%
LINEs:		2,468,426	1,653,598,627	14.12%
	CRE/SLACS	3,647	2,242,901	0.02%
	L2/CR1/Rex	851,669	530,457,586	4.53%
	R1/LOA/Jockey	34,896	14,318,566	0.12%
	R2/R4/NeSL	278,491	183,054,340	1.56%
	RTE/Bov-B	150,980	71,897,444	0.61%
	L1/CIN4	12,271	6,622,898	0.06%
LTR elements:		362,433	317,035,136	2.71%
	BEL/Pao	4,627	5,326,317	0.05%
	Ty1/Copia	2,264	5,727,452	0.05%
	Gypsy/DIRS1	345,898	301,359,837	2.57%
	Retroviral	6,119	1,190,412	0.01%
DNA transposons		1,931,104	724,641,702	6.19%
	hobo-Activator	746,706	280,232,486	2.39%
	Tc1-IS630-Pogo	617,804	247,553,787	2.11%

	PiggyBac	70,298	21,725,655	0.19%
	Tourist/Harbinger	11,518	1,969,450	0.02%
	Other (Mirage, P-element, Transib)	5,972	3,246,023	0.03%
Rolling-circles		131,303	38,104,061	0.33 %
Unclassified:		25,981,963	4,611,039,671	39.38 %
Total interspersed repeats:			7,315,532,371	62.47 %
Small RNA:		123,779	36,746,815	0.31 %
Satellites:		10	8,614	0.00%
Simple repeats:		5,286,058	459,293,181	3.92 %
Low complexity:		250,904	20,431,074	0.17 %

Supplemental Table 5. Fossil calibration information used for the Divergence time estimation in Figure 1a

	Mean Divergence Time (Ma)	95% Confidence Interval [Min., Max.] (Ma)	Fossil Constraints (Ma)	References for Fossil Constraints
Cephalopoda vs. (Bivalvia + Gastropoda) split	530.71	[464.5, 592.0]	549	Grant et al., 1991
Crown Cephalopoda (Nautiloidea vs. Coleoidea split)	421.96	[352.8, 497.4]	408	Kröger & Mapes, 2007
Crown Coleoidea (Decapodiformes vs. Octopodiformes)	255.28	[212.6, 319.5]	240	Schweigert & Fuchs, 2012 Kröger & Mapes, 2007
Crown Decapodiformes (*Doryteuthis* vs. *Thysanoteuthis*)	102.13	[29.8, 225.0]		
Crown Octopodiformes (*Vampyroteuthis* vs. Incirrata)	252.31	[208.4, 316.2]	162	Fuchs & Weis, 2008
Crown Incirrata (Eledone vs. (*Argonauta* + *Octopus*) split)	107.06	[52.5, 204.4]		
Argonauta vs. *Octopus* split	73.22	[33.2, 158.2]	29	Kobayashi, 1954
